# Synaptic neuron-astrocyte communication is supported by an order of magnitude analysis of inositol tris-phosphate diffusion at the nanoscale in a model of peri-synaptic astrocyte projection

**DOI:** 10.1186/s13628-018-0043-3

**Published:** 2018-02-12

**Authors:** Pavel Montes de Oca Balderas, Horacio Montes de Oca Balderas

**Affiliations:** Unit of Dynamic Neurobiology, Neurochemistry Deprtment Instituto Nacional de Neurología y Neurocirugía, Insurgentes Sur #3877, Col. La Fama, C.P. 14269 Ciudad de México, Mexico

**Keywords:** PAP, Neuron-astrocyte communication, Tripartite synapse, Astrocyte, Ca2+ stores

## Abstract

**Background:**

Astrocytes were conceived for decades only as supporting cells of the brain. However, the observation of Ca2+ waves in astrocyte synctitia, their neurotransmitter receptor expression and gliotransmitter secretion suggested a role in information handling, conception that has some controversies. *Synaptic Neuron-Astrocyte metabotropic communication mediated by Inositol tris-phosphate* (SN-AmcIP3) is supported by different reports. However, some models contradict this idea and Ca2+ stores are 1000 ± 325 nm apart from the Postsynaptic Density in the Perisynaptic Astrocyte Projections (PAP’s), suggesting that SN-AmcIP3 is extrasynaptic. However, this assumption does not consider IP3 Diffusion Coefficient (*Dab*), that activates IP3 Receptor (IP3R) releasing Ca2+ from intracellular stores.

**Results:**

In this work we idealized a model of a PAP (PAPm) to perform an order of magnitude analysis of IP3 diffusion using a transient mass diffusion model. This model shows that IP3 forms a concentration gradient along the PAPm that reaches the steady state in milliseconds, three orders of magnitude before IP3 degradation. The model predicts that IP3 concentration near the Ca2+ stores may activate IP3R, depending upon Phospholipase C (PLC) number and activity. Moreover, the PAPm supports that IP3 and extracellular Ca2+ entry synergize to promote global Ca2+ transients.

**Conclusion:**

The model presented here indicates that Ca2+ stores position in PAP’s does not limit SN-AmcIP3.

**Electronic supplementary material:**

The online version of this article (10.1186/s13628-018-0043-3) contains supplementary material, which is available to authorized users.

## Background

The neurocentric theory denied for decades the role of neuroglia on information handling within the Central Nervous System (CNS), believing that neurons were the only type of cell involved in such function [1]. In this scheme, astrocytes were considered supporting cells for neuronal metabolism, survival and function. This conception started to change 25 years ago, when it was reported that astrocytes communicate through Ca2+ waves that travel through their sinctitia [2]. This finding together with astrocyte neurotransmitter receptor expression; secretion of gliotransmitters; and strategic location with the Perisynaptic Astrocyte Projections (PAP’s), set the basis for the Tripartite Synapsis Hypothesis [3]. This idea proposed that astrocytes respond to and regulate neuronal synaptic communication, thus participating in information processing within CNS. Today, a large amount of experimental evidence supports this notion and astrocytes are known to modulate and synchronize synaptic neuronal activity and participate in CNS functions that were thought exclusive of neurons [4–10].

Nonetheless, some results and conclusions in this field are still disputed [11, 12]. The *Synaptic Neuron-Astrocyte metabotropic communication mediated by Inositol tris-phosphate* (IP3) (SN-AmcIP3) that involves Phospho Lipase C (PLC) activation, IP3 synthesis, and IP3 Receptor (IP3R) activation that leads to Ca2+ release from intracellular stores, is disputed. This mechanism is supported by metabotropic Glutamate Receptors (mGluR) in PAP’s observed by light and electron microscopy (EM) [13–15]; PAP activity dependent on mGluR, GTP or IP3 [16–18]; and the observation of Ca2+ stores in PAP’s [8, 19, 20]. However, some groups have challenged this idea because IP3R type 2 KO model present normal neuronal or CNS function [21, 22], despite the reduction of Ca2+ transients in soma and a partial reduction of local Ca2+ activity in astrocyte projections [23]. In addition, PAP’s seem to be devoid of Ca2+ stores [24, 25], and in a recent EM study they were located 1000 ± 325 nm far from its contact with the synapse [26]. Thus it was suggested that SN-AmcIP3 does not occur in PAP’s, but instead in extrasynaptic sites, supporting that Ca2+ increase in PAP’s is given by Ca2+ entry through membrane channels [27].

Nevertheless, this conclusion did not consider the high diffusion coefficient of IP3. Therefore, in an attempt to obtain transport phenomena insights, we implemented a isothermal, dimensionless mass diffusion model utilised in other areas of science and engineering to evaluate the correlation between *Dab* and the boundary conditions under which the diffusing material moves exclusively as a result of a concentration gradient [28]. We utilise geometrical and biophysical parameters for this model reported in the literature to enable an order of magnitude analysis of the mass diffusion process of IP3. With this model we evaluate IP3 diffusion from the membrane contacting a glutamatergic synapse to the putative location of the Ca2+ stores, in an effort to analyse neuron-astrocyte interactions. We find that IP3 forms a concentration gradient along the PAPm that reaches the steady state in milliseconds. The model also predicts that IP3 concentration near the Ca2+ stores achieves a concentration that could activate IP3R in a time dependent upon PLC number and activity. Thus, the biophysical model presented here indicates that SN-AmcIP3 is not limited by Ca2+ stores distance from the synapse. In addition, the model supports that Ca2+ entry through membrane channels and Ca2+ release from stores synergistically elicit Ca2+ waves that self-perpetuate and become global Ca2+ transients.

## Methods

### Perisynaptic astrocyte projection model (PAPm)

**Fig. 1:**
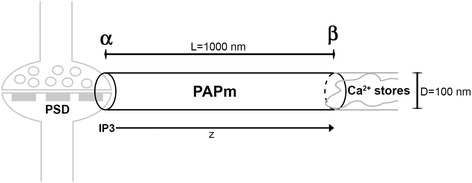
The Perisynaptic Astrocyte Projection model (PAPm). The PAP of an astrocyte is idealized as a cylinder with diamater (D) = 100 nm and length (L) = 1000 nm. The length is the distance between the membrane that contacts the PSD (side **α**) and the location of the Ca2+ stores (side **β**) according to the work by Patrushev et al. [26]. The IP3 is synthesized in α and diffuses gradually inside the cylinder in the **z** axis towards β, forming a gradient that after some time reaches the steady state, when the concentration along the cylinder is almost equal at any point

A PAP model (PAPm) based on reported observations [25] is proposed (Fig. 1). For simplicity, the PAPm is idealized as a cylinder with a length (L) of 1000 nm, corresponding to the average distance measured by Patrushev et al. [26] between the Post Synaptic Density (PSD) contacted by the PAP (the membrane that contacts the synapse; α in Fig. 1), and the Ca2+ stores in the PAP (β in Fig. 1). A diameter (D) of 100 nm is considered, length in the upper limit reported for these structures (50–100 nm) [25]. As it will be described below, radial diffusion is deliberately neglected in a first attempt to carry out an order of magnitude analysis of IP3 diffusion. It must be noted that despite PAP’s are leaf-like structures with a high surface/volume ratio, the actual form of the PAPm is non-relevant since IP3 diffusion is modelled in one dimension, that is, the linear distance between the site of IP3 synthesis and the location of Ca2+ stores [29]. The volume of the cylinder (PAPm) described above will be utilised to describe IP3 diffusion, concentrations and number of molecules as a function of time. The internal volume of this PAPm is V = πD^2^L/4 = 7.8539X10^-21 m^3 or 7.8539X10^-18 l. Table 1 shows the relationship between IP3 concentration and number of IP3 molecules for this volume. Thus, at 1 μM there are approximately 5 IP3 molecules; whereas at ≈0.2 μM concentration there is 1 molecule of IP3. Below this concentration we consider there is no IP3 at all.

**Table 1:** Concentration – molecules relationship in a volume of 7.8539X10^-18 l as per the PAPm structural model

Concentration	Molecules
1 M	4.73E + 06
1 mM	4.73E + 03
1μM	4.73E + 00
1 nM	4.73E-03

According to previous work [30] the IP3 precursor phosphatidyl-inositol di-phosphate (PIP2) is localized at the cell membrane in clusters with a diameter of ≈50–55 nm as measured by STORM microscopy. This value is close to that evaluated by previous studies that found a diameter of 73 nm of these microdomains [31]. Thus, it is possible to conceive that in α such PIP2 clusters are present. Despite this has not been confirmed experimentally, the presence of PIP2 clusters (or the alternative PLC substrate Phosphatidyl inositol tris-phosphate [PIP3]) at the PAP cell membrane near the synapse is clearly a sine qua non condition for SN-AmcIP3. It must be noted that the PAPm base area with 100 nm diameter could contain more than one of these clusters since the area is approximately ≈4 times larger than a single PIP2 cluster area (7854 nm^2^ vs 1963 nm^2^). Moreover, considering the PAP in vivo geometry that surrounds the synapse, this area could be even larger. However, for simplicity, in this work we will consider that there are only three of such clusters at α (Fig. 1) that maintain a constant concentration of PIP2 during the time lapse of interest for this model. That is, that no PIP2 synthesis occurs, but neither that it is depleted by a mechanism other than PLC activity.

One PIP2 cluster or microdomain is predicted to contain ≈1000 PIP2 molecules [30, 31]. Therefore, for the volume of the PAPm calculated above, there are ≈3000 PIP2 molecules in the three clusters, that represent a molar concentration within the PAPm of ≈600 μM. The same concentration could potentially be reached by IP3 after all PIP2 is cleaved by PLC into IP3 and Diacylglycerol (DAG). Given that IP3 is a soluble molecule and may diffuse within the PAP, it is conceivable that after some time it may reach the Ca2+ stores located in β (Fig. 1), and finally reach a steady state concentration within the PAPm. This may occur only if IP3 is not degraded or transformed into another chemical species. In any case, there are only two plausible options: 1) IP3 accumulates once it reaches the Ca2+ stores, reducing the concentration gradient between α and β or 2) it is removed by whichever biochemical transformation mechanism one believes to occur. In the latter case, regardless the detailed mechanism, the rate of IP3 removal clearly contributes to the concentration distribution profile within the cylinder and more particularly to the gradient between opposite ends of the PAPm. In any case, the largest concentration gradient of IP3 along the cylinder occurs when each IP3 molecule is instantaneously removed near the Ca2+ stores. An unexpected but nonetheless logical exception occurs when IP3 generates more IP3 near the Ca2+ stores. At intermediate removal rates, the concentration gradient is smaller and at the limit when no removal occurs or it tends to zero near the Ca2+ stores, IP3 accumulates. This situation in which IP3 accumulates near the Ca2+ stores represents a case in which the concentration gradient between α and β decreases over time, reducing the driving force for IP3 mass diffusion, but IP3 concentration near the stores is maximal. For our model we assume that IP3 accumulates in the time lapse of interest and that the only driving force responsible for the transport of IP3 is a concentration gradient. Other driving forces that could induce IP3 mass transport such as thermal gradients, surface tension gradients, electrical fields, magnetic fields, pressure gradients, elastic deformation of the cylindrical system, etc., are considered negligible. Furthermore, we assume that IP3 velocity can be described by motion in one dimension, z using conventional cylindrical coordinates (Fig. 1).

Also, for this work we assume that at least one PLC molecule is available within the PAPm to perform the synthesis of IP3 from PIP2. Despite the presence of PLC in PAP’s has not been demonstrated experimentally, it is clear that this condition must be fulfilled in order for IP3 signalling to occur in PAP’s. Moreover, we evaluated the model presented here for three different PLC concentrations within the PAPm: 0.2 μM (1 PLC molecule); 2 μM (10 PLC molecules) and 20 μM (100 PLC molecules). Although the average number of PLC molecules per membrane area has been estimated (3/μm^2^) in a cell [32], and such area is larger than the PAPm surface, it is also true that PLC is compartmentalized within cells [33, 34]. Moreover, it has been reported that in astrocytes Ca2+ signalling proteins (including PLC) segregate into lipid raft microdomains [35]. Thus it is possible to assume that PLC molecules are located by intracellular transport mechanisms to specific domains of the membrane, in this particular case to the membrane that contacts the synapse in PAP’s.

To further build the model it is relevant to know the velocity of IP3 synthesis by PLC. We considered the specific activity for PLC-β3 at 30 °C reported by Kadamur and Ross [36] that is > 1000/s, although according to their own experience, the catalysis constant (κ_cat_) could be > 5000/s, depending upon the substrate concentration [36]. Despite in astrocytes PLCβ1 mediate mGluR signalling [37], these specific activity values are useful for our model. Importantly, with these specific activity values, one PLC molecule would cleave all PIP2 and synthesize IP3 in a time below or near the earliest time estimated for IP3 degradation to occur (0.8 s) [38, 39]. Clearly, these PIP2 depletion times would be shorter with larger numbers of PLC or its activity, however, we consider the up- or down-regulatory mechanisms of PLC activity negligible [36]. Nevertheless, the number of PLC molecules considered here for the model and their specific activities give a functional range that we assume comprise these regulatory mechanisms. The values and assumptions for the variables employed in this model are presented in Table 2.

**Table 2:** Variables employed in the PAPm to evaluate the time dependent mass diffusion of IP3 along one dimension

Variable	Value	Reference
PAPm L	1000 nm	Patrushev 2013 [26]
PAPm D	100 nm	Reichenbach 2010 [25]
PAPm volume	7.8539X10^-21 m^3	–
IP3 Dab	300 μm^2^/s	Kang 2009 [41]
IP3 deagradation	0.8 s	Fink 2001 [38]
PIP2 number in PAPm	3000	Van den Bogaart 2011 and Wang 2012 [30, 31]
[IP3] boundary condition	Constant	–
Maximal [IP3] in PAPm	≈600 μM	–
PLC molecules in PAPm	1, 10, 100	–
PLC specific activity	1000/s-5000/s	Kadamur and Ross 2013 [36]

On the other hand, it is known that PIP3 may also be used as PLC substrate to generate IP3 and that it is also concentrated into clusters even larger in size than those of PIP2, although with less concentration (≈1/2–1/6 of PIP2) [30, 31]. However, for simplicity in our model, PIP3 is not considered.

The first physicochemical question that arises with these assumptions is whether *Dab* of IP3 is concentration dependent within the range assumed here for SN-AmcIP3. For simplicity, we will assume constant *Dab*. A second physicochemical question that arises is if at given low concentrations and volumes the continuum theory of mass diffusion is accomplished. However, again for simplicity we will assume that Fick’s law of diffusion is applicable for this analysis (see discussion).

### Mathematical model

For the considerations given above, the simplest transient mathematical model describing mass diffusion in one dimension is given by Crank [28]:

1 $$ \frac{\partial Ca}{\partial t}={D}_{ab}\frac{\partial^2 Ca}{\partial {z}^2} $$

Where *C*_*a*_ is the molar concentration of the solute, *t* is the time, *Dab* the diffusion coefficient of the solute in the cytoplasm and *z* is the coordinate axis along the cylinder of length *L*.

For simplicity and in order to generate a general solution that enables analysis of multiple geometrical and physical situations such as initial concentrations and mass diffusion coefficients, Eq. 1 can be re-written in terms of dimensionless coordinates as:

2 $$ \frac{\partial \phi }{\partial r}=\frac{\partial^2\phi }{\partial {\eta}^2} $$

where *ϕ*=*(C*_*a*_*-C*_*0*_*)/(C*_*1*_*-C*_*0*_*)* is the dimensionless molar concentration, *τ=t Dab /L*^*2*^ is the dimensionless time and *η=z/L* the dimensionless length of the cylinder. *C*_*1*_ is the concentration of solute at *η=0* i.e. at α and *C*_*0*_ is the initial constant concentration of solute in the PAPm.

The general solution of Eq. 2 including a number of special cases can be found elsewhere [40], including one of the simplest cases of interest in which the following set of initial and boundary condition are utilised:

Initial conditions: *ϕ=*0 at *τ*=0 for every *η*> 0.

Boundary condition 1: *ϕ*=1 at *η*=0 for every *τ*≥0.

Boundary condition 2: $\frac{\mathrm{\partial \upphi }}{\mathrm{\partial \upeta }}=0$ at *η*=1, for every *τ*≥0.

3 $$ \phi \left(\tau, \eta \right)=1-\frac{4}{\pi}\sum \limits_{n=0}^{\infty}\left[\left(\frac{{\left(-1\right)}^n}{2n+2}\right){{e^{-\left(2n+1\right)}}^2}^{\pi^2}\raisebox{1ex}{$\tau $}\!\left/ \!\raisebox{-1ex}{$4$}\right.\left(\cos \frac{\left(2n+1\right)\pi \eta}{2}\right)\right] $$

These conditions represent an ideal situation in which initially (*τ*=0) there is no solute inside the cylinder *ϕ*(*τ*=0,*η*> 0) = 0, situation that perhaps hardly occurs in astrocytes in vivo. An improved model would consider a more realistic set of initial conditions of the cell. Boundary condition 1 indicates that the concentration of solute is constant at all times at the base of the PAPm i.e. *ϕ*(*τ*≥0,*η*=0) = 1. An improved model would consider a spatial distribution of the concentration at α i.e. *ϕ*(*τ*≥0,*η*=0) = f(*τ*, *ρ*, *θ*, *η*=0) where *ρ* and *θ* are the other two dimensionless coordinates in a cylindrical coordinate system. Boundary condition 2 indicates that at *η*=1 the solute accumulates over time as concentration changes with respect to the dimensionless length *η* i.e. at *η*=1, $\frac{\mathrm{\partial \upphi }}{\mathrm{\partial \upeta }}=0$. A further improved model could include a reaction kinetics model to account for the removal of IP3 near the Ca2+ stores. However, for the purpose of this model IP3 removal is not considered since the time lapse of interest is below the earliest time predicted for IP3 degradation to occur, as explained above. The reader interested in a more complex biophysical model at the microscale level that considers this and other variables is referred to previous works and references therein [39, 41].

The above described dimensionless mathematical model enables the description of a normalised system independent of the absolute values of solute concentration, cylinder length and time, in which the physical parameters, *C*_*a*_, *Dab* and *L* are hidden in the dimensionless concentration (*ϕ*), length (*η*) and time (*τ*), and therefore is valid for any combination of these parameters including the values of *C*_*1*_ and *C*_*0*_. It is of our particular interest to analyse the mass diffusion process at the nanoscale because only a handful of molecules are involved at μM concentrations for the PAP scale. Moreover, it is of our interest to estimate how fast IP3 diffuses from α to β within the PAPm, and how IP3 concentration develops inside the PAPm.

As can be seen, the model proposed is very simple. However for a dilute system in which interactions between IP3 molecules can be considered negligible and *Dab* is independent of IP3 concentration, the proposed model provides a basic platform to rationalise motion of IP3 at the nanoscale from any region in space to any point in space in its most immediate environment. It is within this space where a critical mass diffusion process of IP3 is assumed to occur, responsible for Ca2+ release from intracellular stores. It is the simplicity of the model the basis for our discussion on the relationship between signalling processes and the cellular structure where these processes occur.

## Results

### IP3 concentration gradient within the PAPm

**Fig. 2:**
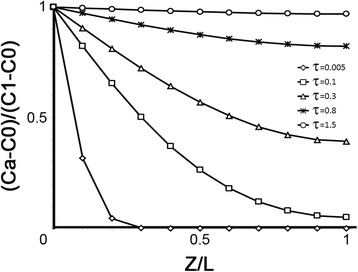
Dimensionless gradient profiles in the PAPm. Concentration distributions at various dimensionless time values (τ) in a cylinder with initial uniform concentration C1 at the base C0 concentration at the opposite end. *τ=Dabt/L*^*2*^

The dimensionless IP3 concentration distributions at various dimensionless times (represented by parameter *τ*) in the PAPm depicted in the previous section were computed and the resulting profiles are shown in Fig. 2. As can be seen, for each *τ* value there is a gradient in the concentration between α (Z/L = 0) and β (Z/L = 1). As *τ* increases this gradient diminishes and at larger *τ* values (τ = 1.5) the gradient is almost zero, when practically the concentration in the cylinder is nearly constant and equal to concentration at Z/L = 0, reaching almost the steady state, when no more or only small changes in substance concentration are observed if *τ* increases. Thus, according to these profiles, with *τ* = 0.1 the IP3 gradient would have reached the opposite side of the cylinder, that is, IP3 molecules had reached the Ca2+ stores.

Given that.

$$ t= tL\hat{\mkern6mu} 2/ Dab. $$

thus the same profiles of concentration gradients are potentially reached by different solutes for the same PAPm in times dependent upon their *Dab*.

### Number of IP3 molecules in the vicinity of PAPm Ca2+ stores

**Fig. 3:**
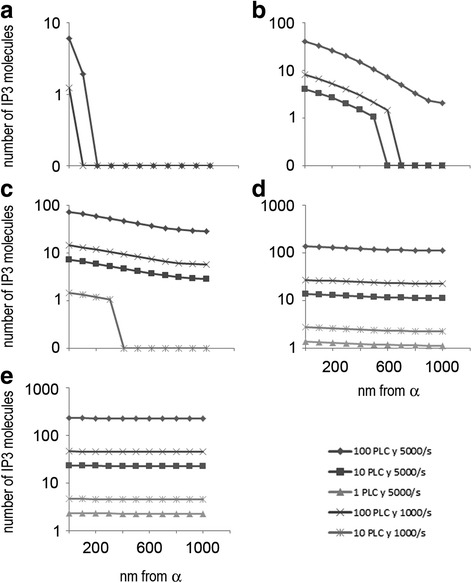
Approximate number of IP3 molecules along the PAPm for different PLC conditions and times, according to the concentration profiles depicted in Fig. 2. **a** 16 μs**; b** 333 μs; **c** 1 ms; **d** 2.6 ms; **e** 5 ms

In the framework of neurobiology, the numerical solution given above for Eq. 2 is interesting when it is interpreted in actual IP3 numbers within the PAPm and time values, since it offers an insight into what may happen with PLC metabotropic signalling in an actual PAP. Since the area under each concentration profile is proportional to the total amount of substance diffused into the cylinder at a particular time point and the total number of IP3 molecules in the PAPm at a given time depends on the number of PLC molecules and their specific activity, then, it is possible to estimate the number of IP3 molecules for different time values given by t=*τ* L^2/*Dab*. The numerical solution for this approximation (Additional file 1: Tables S1) for t = 16.7 μs, 333 μs, 1 ms, 2.6 ms and 5 ms (that result from each τ value) was calculated for different PLC numbers and specific activities as described in the model section (1, 10 and 100 PLC molecules with specific activity 1000/s or 5000/s). We plotted this data into profiles of the number of IP3 molecules in the PAPm that are shown in Fig. 3. It must be noted that since it is not possible to have fractional IP3 molecules as it is solved by the numerical solution, we rounded up or down the number of IP3 molecules for these plots. Moreover, for this same reason we only show in Fig. 3 the profiles with solutions ≥1 that is, in which at least one IP3 molecule is in the given Z position for the given PLC and t parameters.

With this approximation we found that, as shown in Fig. 3a, in only 16.7 μs after PLC activation with 100 PLC at 5000/s, ≈2 IP3 molecules would diffuse ≈100 nm within the cylinder with 6 molecules near α. With 100 PLC at 1000/s, only one IP3 molecule would be synthesized and located near α. Interestingly, after 333 μs (Fig. 3b), with 100 PLC at 5000/s, the IP3 gradient would have reached β, but only ≈2 IP3 molecules would be in its vicinity. With these conditions, ≈41 IP3 molecules would be near α and the rest of the IP3 molecules synthesized would be distributed along the gradient in the PAPm. In contrast, with 10 PLC at 5000/s in 333 μs, the IP3 gradient would reach only 600 nm, with ≈8 IP3 molecules near α. A similar situation would occur with 100 PLC at 1000/s in 333 μs, since the gradient would reach only 500 nm and ≈4 IP3 molecules near α. After 1 ms (Fig. 3c) and three PLC numbers the IP3 gradient would reach the vicinity of Ca2+ stores. With 100 PLC at 5000/s ≈ 29 IP3 molecules would be near β; with 100 PLC at 1000/s it would be ≈6 IP3 molecules, and with 10 PLC at 5000/s it would be ≈3 IP3 molecules. After 2.6 ms (Fig. 3d), the gradients would almost reach an homogeneous distribution along the PAPm. With 100 PLC at 5000/s ≈ 112 IP3 molecules would be near β, whereas ≈22 and ≈11 would be there at 100 PLC at 1000/s and 10 PLC at 5000/s, respectively. Notably at this time point, with only one PLC at 5000/s ≈ 1 IP3 molecule would be near the Ca2+ stores, whereas with 10 PLC at 1000/s there would be ≈2. Finally, after 5 ms (Fig. 3e) the gradients would be closer to the steady state and the number of IP3 molecules in the vicinity of β would be between ≈225 for 100 PLC at 5000/s and ≈2 for 1 PLC at 5000/s. Interestingly, with 1 PLC at 1000/s, that is with 5 IP3 molecules synthesized, the model predicts that only fractional IP3 molecules would be distributed along the PAPm (not shown). This is a non-logical situation since, as explained above, it is not possible to have fractions of IP3 molecules, and therefore the 5 molecules synthesized would be distributed in a different manner that is not possible to predict with the model used here. The number of IP3 molecules in the vicinity of Ca2+ stores (β) in the PAPm are summarized for the PLC conditions in Table 3. This data indicates that IP3 generated in the membrane of the astrocyte contacting the synapse reaches the Ca2+ stores in a time scale of μs- ms, accumulating there in high quantities in few ms.

**Table 3:** Summary of the approximate number of IP3 molecules in the PAPm and near the Ca2+ stores (β) with different conditions of PLC number, specific activity and time

Condition	Number of IP3 within the PAPm	Number of IP3 near β
100 PLC, 5000/s, 333 ms	167	2
100 PLC, 5000/s, 1 ms	500	29
10 PLC, 5000/s, 1 ms	50	3
100 PLC, 1000/s, 1 ms	100	6
100 PLC, 5000/s, 2.6 ms	1335	112
10 PLC, 5000/s, 2.6 ms	133	11
1 PLC, 5000/s, 2.6 ms	13	1
100 PLC, 1000/s, 2.6 ms	267	22
10 PLC, 1000/s, 2.6 ms	27	2
100 PLC, 5000/s, 5 ms	2500	225
10 PLC, 5000/s, 5 ms	250	22
1 PLC, 5000/s, 5 ms	25	2
100 PLC, 1000/s, 5 ms	500	45
10 PLC, 1000/s, 5 ms	50	4

## Discussion

According to the model for IP3 diffusion at the PAPm nanoscale proposed here, the lack of Ca2+ stores in PAP’s near the PSD does not rule out SN-AmcIP3 and suggests that PLC number and activity is the limiting step. This conclusion assumes that the molecular signalling machinery is present in the PAP. In this regard, mGluR3 and 5 have been localized in PAP’s by EM and mGluR1 by fluorescence microscopy [13–15, 37]. Despite astrocyte and PAP responses depend upon mGluR signalling, their expression of mGluR5 declines with age, mGluR1 seems to be only slightly involved and mGluR3 use the adenylate cyclase pathway [20, 29, 37, 42]. Therefore, it is possible that other metabotropic receptors could mediate SN-AmcIP3 in adult animals [43]. Interestingly, we found a metabotropic-like flux independent NMDAR in cultured astrocytes that could function in tissue astrocytes [44]. Also in astrocytes, different PLC isoforms have been observed and the molecular machinery that mediates Ca2+ release from intracellular stores is located in raft-like microdomains [35, 45]. Moreover, supramolecular ensembles of signalling molecules that include PLC are organized into specific sites of the membrane where they optimize the speed of signal transduction [46]. Thus, it is possible to conceive that the molecular machinery responsible for metabotropic signalling through IP3 is present in PAP’s, unless a specific mechanism prevents it.

Interestingly, the lack of Ca2+ stores in PAP’s may be related with the latency observed in astrocyte’s responses or secretion [47], slower than neuronal responses, where Ca2+ stores are closer (≈500 nm) to the synapse [26]. Therefore, the lack of Ca2+ stores supports the idea that PAP’s are subcellular specializations [7], if not experimental artefacts as discussed elsewhere [8].

In this work we analyse a range of different conditions regarding the number and activity of PLC, in an effort to include different functional conditions that may regulate both variables. The model shows that under different PLC conditions (Table 3), 2–225 IP3 molecules would reach the Ca2+ stores in ≤5 ms. These numbers could be underestimated because PLC specific activity considered here was measured at 30 °C [36] and the PAP could have more PIP2 microdomains than three. With few IP3 molecules near the Ca2+ stores the activation of IP3R would have low probability to occur, according to a recent model of IP3R activation (see below) [48], although the longer the time the larger the probability. On the other hand, despite IP3 diffusion in tissue PAP’s most probably does not occur in a single dimension as modelled here, the order of magnitude analysis presented here sets up a useful temporal framework for this phenomenon that further allows its dissection and analysis.

The mathematical model gives a numerical solution set that does not necessarily fits a real representation in the biological context, this occurs with fractional numbers of IP3. In addition, when low IP3 synthesis results by low PLC number and/or specific activity, it is questionable whether the Fick’s law of mass diffusion accomplishes, as described above. In this scenario, the motion of IP3 would have no single direction in the Z axis, since it is given by the IP3 gradient concentration itself, and with few IP3 molecules the gradient is not continuous. In such conditions, the model presented here is unable to predict the behaviour of IP3 molecules due to mass diffusion.

With the approximate numbers of IP3 near the Ca2+ stores (Table 3), two questions arise that are relevant in the context of a PAP. The first is whether the number of PLC molecules assumed here is close to that occurring in an actual PAP. To analyse this question we recall the estimations made for M_1_ muscarinic Receptors (M_1_R) [32], suggesting that there are 40 Gq proteins and 10 PLC molecules per each endogenous M_1_R in the membrane. To our knowledge, these estimations have not been made for mGluR, but if the proportion is maintained, then the number of PLC molecules in PAP’s must be around 10 if one mGluR is present, or more if there are more mGluR, condition that is plausible to occur. Therefore, the number of PLC molecules used for this analysis (1, 10 and 100) constitutes a range that likely includes the number occurring in an actual PAP.

The second question is how many IP3 molecules are required to activate an IP3R. In a cell, 100 nM IP3 evokes a half-maximal Ca2+ response by IP3R [38], but in the PAPm 1 IP3 = 200 nM, and this concentration would be higher in a leaf-like PAP. Then, this IP3 threshold at the microscale is meaningless at the nanoscale of a PAP. In this regard, it would be interesting to assess theoretically and experimentally the PAP IP3 threshold to elicit local or global Ca2+ responses (see below). It has been shown that IP3R requires four IP3 to be activated [48]. Thus, at least four IP3 are required near the Ca2+ stores to activate one of its IP3R. However, according to the work by Alzayady et al. [49] and its implications, IP3 sites in IP3R can buffer IP3 without receptor activation. Therefore, unless a single IP3R is at the Ca2+ stores membrane, with 4 IP3 molecules Ca2+ release has low probability to occur, and the more IP3R the less probable.

Here then, the density of IP3R in intracellular Ca2+ stores is a relevant question, that to our knowledge has not been explored. However, we can get an approximation considering the area of IP3R in the plane of the membrane (≈380 nm2, Protein Data Bank: 3jav) and the area of a transversal section of the PAPm (7583 nm2) that would be close to the membrane area of the Ca2+ stores, although its geometry could increase it. Then, a maximum of ≈20 IP3R could be at the Ca2+ stores facing the PAP, however, since other molecules and Ryanodine Receptors (RyR) must also be there, we estimate that ≈10 IP3R face the PAPm, close to the number of IP3R required to generate a Ca2+ puff [50, 51]. Importantly, RyR perpetuates the Ca2+ transient through Ca2+ Induced Ca2+ Release (CICR) and although IP3R may also participate, it conducts three times more Ca2+, however their participation varies in astrocytes of different origin [52–54]. Then, ≈40 IP3 molecules could potentially activate the full pool of IP3R. However, given the nature of molecular interactions, IP3 diffusion and IP3R activation, this number could probably activate only some of the IP3R resulting in a sub-maximal release of Ca2+. In addition, IP3-IP3R binding kinetics would contribute to determine the number of IP3R opened and the amount of Ca2+ released, that in turn would facilitate IP3R function (see below). Nevertheless, since IP3R release of Ca2+ is graded, then the more IP3 the higher probability that IP3R are opened and the more Ca2+ is released. These considerations are consistent with the linear relationship between synaptic activity and astrocyte’s Ca2+ activity [43], and further question how many IP3R must be activated to release enough Ca2+ to generate a response that self-perpetuates. Importantly, the leaf-like geometry of a tissue PAP would significantly reduce its transversal area, and consequently the number of IP3R in the Ca2+ store facing the PAP could be less than those estimated here.

Ca2+ activity in brain astrocytes has been categorized spatially as local (microdomains) or global (somatic) [18, 23]. Local events are restricted to projections and its main component is Ca2+ entry through membrane channels, although IP3R2 also participates, whereas the frequency of global events critically depends upon IP3R2, although its KO does not fully eliminate them [23, 43, 55, 56]. Our PAPm supports that Ca2+ entry through membrane channels and Ca2+ release through IP3R cooperate to elicit local Ca2+ activity and that Ca2+ release through IP3R is important to trigger global activity. This is because IP3 high *Dab* (≈300 μm^2^/s) enables “long” range activity at Ca2+ stores, whereas Ca2+ small *Dab* (≈30 μm^2^/s) [39] constrains its diffusion, and its entry would rather regulate events in the “short” range near the membrane, such as PLC activation or actin remodelling among others. In this scenario the Ca2+ dependence of IP3R becomes a relevant matter since both IP3R agonists (IP3 and Ca2+) delivered from the PAP membrane opposing the synapse would not reach he Ca2+ store simultaneously. Interestingly, IP3R2 expressed in astrocytes may work at low Ca2+ levels as those in astrocyte basal conditions (50–100 nM) [53]. Thus, IP3 “alone” could potentially trigger Ca2+ release that could further potentiate IP3R response itself and activate RyR and start CICR. Here it is important to note that CICR through IP3R requires IP3and Ca2+, whereas Ca2+ alone is able to start CICR through RyR, thus suggesting a main role of RyR in global responses. This is because diffusion of IP3 beyond the PAPm would be constrained by its amount synthesized and by the PAP compartmentalization, although again, it has been observed that IP3R and RyR may play different roles in astrocytes of different origin [54]. Interestingly, this constrain could be overcome when activity from different PAPs is integrated, therefore eliciting a global response, scenario that is in agreement with experimental observations in which global responses require high neuronal firing [43].

Recently, a novel work suggested that IP3 D*ab* is ≈30 times smaller (10 μm2/s) of that estimated previously (300 μm2/s) [57]. This estimation is based on the assumption that immobile IP3 binding sites (silent IP3R) present within the cell, not considered in the previously, would hinder IP3 diffusion. Despite this premise is fairly reasonable, this work has some caveats and their conclusion needs further experimental testing, enabling us to stick to the previous IP3 D*ab* value. First, in the work by Dickinson et al. (2016) the role of RyR is not ruled out and some of their observations and experimental settings hint its participation. Therefore, it is possible that their estimation involves the displacement of the Ca2+ wave mediated by RyR and is not only given by IP3 diffusion. Second, in this work a degradation resistant analog of IP3 was used (i-IP3). The chemical structure of this molecule is different of that of IP3 as it contains a additional acetonide group in carbons 2 and 3 instead of hydroxyl groups. According to theories of diffusion in binary liquids and the Stokes-Einstein eq. [58], the diffusion coefficient is inversely proportional to the size of the molecule. Thus, diffusivity of IP3 must not be necessarily equal to the more bulkier i-IP3. In addition, the assumption that immobile IP3 binding sites would hinder IP3 diffusion does not apply to our model because according to the work of Patrushev et al. [26] this PAP region lacks Ca2+ stores and thus silent IP3R. Finally, despite the IP3 D*ab* were 10 μm2/s, the time to reach the gradients presented in Fig. 3 would still be well below (≤ 150 ms) the time of IP3 degradation.

Interestingly, Glu *Dab* in the extracellular space is 150–570 μm^2^/s depending upon different variables [59]. Therefore the Glu to IP3 exchange at the PAP membrane as the signalling mediator would not necessarily suppose a faster diffusion of the signal, but instead a spatial restriction for its diffusion within the PAP. In order to reach extrasynaptic sites close to Ca2+ stores, Glu would have to escape from the synapse and the PAP surrounding it, with a non-directed diffusion. Thus the Glu to IP3 relay would represent an effective and optimal mean for inter-and intracellular signalling. An additional variable not considered in our model is that the number of IP3 molecules synthetized would be related to the amount of Glu released by synaptic activity. It has been demonstrated that the higher the synaptic stimulation the more Glu is secreted [43], and therefore it is reasonable that more IP3 would be synthesized that in turn could reach distant places. In the case of a train of stimulus, the amount of IP3 could reach maximal levels within the PAP and would be limited by the available pool of IP3 precursors and the time required to replenish them. This condition would be irrelevant in the case of low synaptic stimulation. Accordingly, the level of synaptic activity (low or high) is proportional to the Ca2+ response in the astrocyte [43]. Importantly, with high trains of stimulation Glu could spill over and reach extrasynaptic sites where it would activate mGluR.

Astrocytes have been proposed as integrators of synaptic information [60]. This notion is supported by the stimulation of neuronal populations that elicit PAP local Ca2+ events and the increase of global events. In addition, local Ca2+ events are 2–8 times more frequent than global events [23, 43]. These observations, together with the role of Ca2+ entry and Ca2+ release on local activity, let speculate that an integrative mechanism also occurs at individual astrocyte projections. This putative mechanism could be the gradual accumulation of Ca2+ (and/or other molecules) given by the entry and perhaps the release of small amounts of Ca2+ (and/or other molecules i.e. IP3) caused by cycles of suboptimal stimulation, that after some reverberations elicit a global Ca2+ transient through RyR. This mechanism could be related with observations in the IP3R2 KO model, in which global Ca2+ activity is reduced but not absent [23, 43]. If this is true, it is possible to speculate that PAP’s are Ca2+ (or molecular) capacitors, a useful conception for neuron-astrocyte communication modelling, and its cyclic Ca2+ activity could be related with the neuronal synchronization by astrocytes [61, 62].

## Conclusions

In this work, we idealized a model of a PAP to perform an order of magnitude analysis of IP3 diffusion using a transient mass diffusion model. Our results show that a) IP3 forms a concentration gradient along the PAPm that reaches the steady state in milliseconds; b) that IP3 concentration near the Ca2+ stores may activate IP3R depending upon PLC number and activity; and more importantly, c) it supports that IP3 and extracellular Ca2+ entry synergize to generate global Ca2+ transients. Thus, the PAPm and the order of magnitude analysis presented here indicate that SN-AmcIP3 is not limited by the distance measured between the Ca2+ stores in PAP’s.

## Additional file

Additional file 1: Tables S1. The numerical solution for equation 2 for t=16.7 ms, 333ms, 1 ms, 2.6 ms and 5 ms (that result from each t value), calculated for different PLC numbers and specific activities as described in the model section (1, 10 and 100 PLC molecules with specific activity 1000/s or 5000/s). (PDF 231 kb)

## Abbreviations

CICR: Ca2+ Induced Ca2+ Release
CNS: Central Nervous System
D: diameter
*Dab*: Diffusion Coefficient
DAG: Diacylglycerol
EM: Electron Microscopy
Glu: Glutamate
IP3: Inositol tris-phosphate
IP3R: IP3 Receptor
M_1_R: M_1_ muscarinic Receptors
MGluR: Metabotropic Glutamate Receptor
PAP: Perisynaptic Astrocyte Projection
PAPm: Perisynaptic Astrocyte Projection model
PIP2: phosphatidyl-inositol di-phosphate
PIP3: Phosphatidyl inositol tris-phosphate
PLC: Phospholipase C
PSD: Post-Synaptic Density
RyR: Ryanodine Receptors
SN-AmcIP3: Synaptic Neuron-Astrocyte metabotropic communication mediated by IP3
