# Supplementary material for: Synaptic neuron-astrocyte communication is supported by an order of magnitude analysis of inositol tris-phosphate diffusion at the nanoscale in a model of peri-synaptic astrocyte projection
Source: BMC Biophys. 2018 Feb 12;11:3. doi: 10.1186/s13628-018-0043-3 (PMC5809920; doi:10.1186/s13628-018-0043-3)
Supplement: Supplementary file 1 — The numerical solution for equation 2 for t=16.7 ms, 333ms, 1 ms, 2.6 ms and 5 ms (that result from each t value), calculated for different PLC numbers and specific activities as described in the model section (1, 10 and 100 PLC molecules with specific activity 1000/s or 5000/s). (PDF 231 kb) [file 13628_2018_43_MOESM1_ESM.pdf]

IP3 #	8.33333333	0.83333333	0.08333333	1.66666667	0.16666667	0.01666667
			16.7 us			
Z/L	**100 PLC & 5000/s**	**10 PLC & 5000/s**	**1 PLC & 5000/s**	**100 PLC & 1000/s**	**10 PLC & 1000/s**	**1 PLC & 1000/s**
**1**	1.00E-06	1.00E-06	1.00E-06	1.00E-06	1.00E-06	1.00E-06
**0.9**	1.00E-06	1.00E-06	1.00E-06	1.00E-06	1.00E-06	1.00E-06
**0.8**	1.00E-06	1.00E-06	1.00E-06	1.00E-06	1.00E-06	1.00E-06
**0.7**	1.00E-06	1.00E-06	1.00E-06	1.00E-06	1.00E-06	1.00E-06
**0.6**	1.00E-06	1.00E-06	1.00E-06	1.00E-06	1.00E-06	1.00E-06
**0.5**	1.00E-06	1.00E-06	1.00E-06	1.00E-06	1.00E-06	1.00E-06
**0.4**	0.00061145	6.1145E-05	6.1145E-06	0.00012229	1.2229E-05	1.2229E-06
**0.3**	0.01650923	0.00165092	0.00016509	0.00330185	0.00033018	3.3018E-05
**0.2**	0.27821104	0.0278211	0.00278211	0.05564221	0.00556422	0.00055642
**0.1**	1.94013987	0.19401399	0.0194014	0.38802797	0.0388028	0.00388028
**0**	6.11452841	0.61145284	0.06114528	1.22290568	0.12229057	0.01222906

IP3 #	166.666667	16.6666667	1.66666667	33.3333333	3.33333333	0.33333333
			333 us			
Z/L	**100 PLC & 5000/s**	**10 PLC & 5000/s**	**1 PLC & 5000/s**	**100 PLC & 1000/s**	**10 PLC & 1000/s**	**1 PLC & 1000/s**
**1**	2.05465498	0.2054655	0.02054655	0.410931	0.0410931	0.00410931
**0.9**	2.35454545	0.23545455	0.02354545	0.47090909	0.04709091	0.00470909
**0.8**	3.27853231	0.32785323	0.03278532	0.65570646	0.06557065	0.00655706
**0.7**	4.91171961	0.49117196	0.0491172	0.98234392	0.09823439	0.00982344
**0.6**	7.35542169	0.73554217	0.07355422	1.47108434	0.14710843	0.01471084
**0.5**	10.7109529	1.07109529	0.10710953	2.14219058	0.21421906	0.02142191
**0.4**	15.0512596	1.50512596	0.1505126	3.01025192	0.30102519	0.03010252
**0.3**	20.364184	2.0364184	0.20364184	4.0728368	0.40728368	0.04072837
**0.2**	26.5362541	2.65362541	0.26536254	5.30725082	0.53072508	0.05307251
**0.1**	33.356736	3.3356736	0.33356736	6.67134721	0.66713472	0.06671347
**0**	40.5257393	4.05257393	0.40525739	8.10514786	0.81051479	0.08105148

IP3 #	500	50	5	100	10	1
			1 ms			
Z/L	**100 PLC & 5000/s**	**10 PLC & 5000/s**	**1 PLC & 5000/s**	**100 PLC & 1000/s**	**10 PLC & 1000/s**	**1 PLC & 1000/s**
**1**	28.7557226	2.87557226	0.28755723	5.75114452	0.57511445	0.05751145
**0.9**	29.2969036	2.92969036	0.29296904	5.85938071	0.58593807	0.05859381
**0.8**	30.9131331	3.09131331	0.30913133	6.18262663	0.61826266	0.06182627
**0.7**	33.5605318	3.35605318	0.33560532	6.71210636	0.67121064	0.06712106
**0.6**	37.1879068	3.71879068	0.37187907	7.43758136	0.74375814	0.07437581
**0.5**	41.7001858	4.17001858	0.41700186	8.34003715	0.83400372	0.08340037
**0.4**	46.9876699	4.69876699	0.4698767	9.39753397	0.9397534	0.09397534
**0.3**	52.9260337	5.29260337	0.52926034	10.5852067	1.05852067	0.10585207
**0.2**	59.3763255	5.93763255	0.59376326	11.8752651	1.18752651	0.11875265
**0.1**	66.1630271	6.61630271	0.66163027	13.2326054	1.32326054	0.13232605
**0**	73.1325601	7.31325601	0.7313256	14.626512	1.4626512	0.14626512

IP3 #	1250	125	12.5	250	25	2.5
			2.6 ms			
Z/L	**100 PLC & 5000/s**	**10 PLC & 5000/s**	**1 PLC & 5000/s**	**100 PLC & 1000/s**	**10 PLC & 1000/s**	**1 PLC & 1000/s**
**1**	112.267284	11.226728	1.1226728	22.4534569	2.2453456	0.2245345
**0.9**	112.567355	11.256735	1.1256735	22.513471	2.2513471	0.2251347
**0.8**	113.453927	11.345392	1.1345392	22.6907854	2.2690785	0.2269078
**0.7**	114.899721	11.489972	1.1489972	22.9799442	2.2979944	0.2297994
**0.6**	116.877458	11.687745	1.1687745	23.3754917	2.3375491	0.2337549
**0.5**	119.332581	11.933258	1.1933258	23.8665161	2.3866516	0.2386651
**0.4**	122.21053	12.221053	1.2221053	24.4421059	2.4442105	0.2444210
**0.3**	125.443107	12.544310	1.2544310	25.0886214	2.5088621	0.2508862
**0.2**	128.934837	12.893483	1.2893483	25.7869673	2.5786967	0.2578696
**0.1**	132.61752	13.261752	1.3261752	26.523504	2.6523504	0.2652350
**0**	136.39568	13.639568	1.3639568	27.2791361	2.7279136	0.2727913

IP3 #	2500	250	25	500	50	5
			5 ms			
Z/L	**100 PLC & 5000/s**	**10 PLC & 5000/s**	**1 PLC & 5000/s**	**100 PLC & 1000/s**	**10 PLC & 1000/s**	**1 PLC & 1000/s**
**1**	224.533131	22.4533131	2.24533131	44.9066261	4.49066261	0.44906626
**0.9**	224.602674	22.4602674	2.24602674	44.9205348	4.49205348	0.44920535
**0.8**	224.880849	22.4880849	2.24880849	44.9761697	4.49761697	0.4497617
**0.7**	225.321291	22.5321291	2.25321291	45.0642583	4.50642583	0.45064258
**0.6**	225.924003	22.5924003	2.25924003	45.1848005	4.51848005	0.45184801
**0.5**	226.665801	22.6665801	2.26665801	45.3331602	4.53331602	0.4533316
**0.4**	227.523506	22.7523506	2.27523506	45.5047011	4.55047011	0.45504701
**0.3**	228.497116	22.8497116	2.28497116	45.6994233	4.56994233	0.45699423
**0.2**	229.563452	22.9563452	2.29563452	45.9126903	4.59126903	0.4591269
**0.1**	230.676149	23.0676149	2.30676149	46.1352299	4.61352299	0.4613523
**0**	231.812028	23.1812028	2.31812028	46.3624057	4.63624057	0.46362406
